# CD11b Regulates Fungal Outgrowth but Not Neutrophil Recruitment in a Mouse Model of Invasive Pulmonary Aspergillosis

**DOI:** 10.3389/fimmu.2019.00123

**Published:** 2019-02-04

**Authors:** Daniel Teschner, Anna Cholaszczyńska, Frederic Ries, Hendrik Beckert, Matthias Theobald, Stephan Grabbe, Markus Radsak, Matthias Bros

**Affiliations:** ^1^Department of Hematology, Medical Oncology and Pneumology, University Medical Center of the Johannes Gutenberg University, Mainz, Germany; ^2^Department of Dermatology, University Medical Center of the Johannes Gutenberg University, Mainz, Germany; ^3^Department of Pulmonary Medicine, University Medical Center Essen—Ruhrlandklinik, Essen, Germany

**Keywords:** *Aspergillus fumigatus*, pneumonia, β2 integrins, CD11b, complement receptor 3, polymorphonuclear neutrophils, CC-chemokine ligand 5, phagocytosis

## Abstract

ß2 integrin receptors consist of an alpha subunit (CD11a-CD11d) and CD18 as the common beta subunit, and are differentially expressed by leukocytes. ß2 integrins are required for cell-cell interaction, transendothelial migration, uptake of opsonized pathogens, and cell signaling processes. Functional loss of CD18—termed leukocyte-adhesion deficiency type 1 (LAD1)—results in an immunocompromised state characterized by frequent occurrence of severe infections. In immunosuppressed individuals *Aspergillus fumigatus* is a frequent cause of invasive pulmonary fungal infection, and often occurs in patients suffering from LAD1. Here, we asked for the importance of CD11b/CD18 also termed MAC-1 which is required for phagocytosis of opsonized *A. fumigatus* conidia by polymorphonuclear neutrophils (PMN) for control of pulmonary *A. fumigatus* infection. We show that CD11b^−/−^ mice infected with *A. fumigatus* were unaffected in long term survival, similar to wild type (WT) mice. However, bronchoalveolar lavage (BAL) performed 1 day after infection revealed a higher lung infiltration of PMN in case of infected CD11b^−/−^ mice than observed for WT mice. BAL derived from infected CD11b^−/−^ mice also contained a higher amount of leukocyte-attracting CCL5 chemokine, but lower amounts of proinflammatory innate cytokines. In accordance, lung tissue of *A. fumigatus* infected CD11b^−/−^ mice was characterized by lower cellular inflammation, and a higher fungal burden. In agreement, CD11b^−/−^PMN exerted lower phagocytic activity on serum-opsonized *A. fumigatus* conidia than WT PMN *in vitro*. Our study shows that MAC-1 is required for effective clearance of *A. fumigatus* by infiltrating PMN, and the establishment of an inflammatory microenvironment in infected lung. Enhanced infiltration of CD11b^−/−^ PMN may serve to compensate impaired PMN function.

## Introduction

*Aspergillus fumigatus* is a common saprophytic fungus in the environment and is usually well controlled in healthy individuals. However, in patients with immune deficiency e.g., due to chemotherapeutic treatment of malignant diseases or immunosuppressive therapy after allogeneic hematopoietic stem cell or organ transplantation *A. fumigatus* causes invasive pulmonary aspergillosis (IPA) which is highly associated with relevant morbidity and mortality (1, 2). Despite the clinical use of potent antifungal drugs for prophylaxis and treatment of invasive fungal disease IPA still continues to be a highly relevant health issue in the daily clinical care with regard to morbidity, mortality, diagnostic challenges, and costs (3). Polymorphonuclear neutrophils (PMN) play a very important role in the innate host defense against *A. fumigatus* by sufficiently killing outgrowing *A. fumigatus* conidia and hyphae. The crucial importance of PMN in this setting is also reflected by the fact that neutropenia is one major risk factor for the development of IPA (4). While the size of hyphae may prevent the fungus from phagocytosis by PMN, hyphal damage is caused by other PMN effector mechanisms, including the formation of neutrophil extracellular traps (NET) (5). In this setting, the oxidative PMN effector functions are essential for survival of IPA (6). In addition, also monocytes and macrophages substantially contribute to the regulation of antifungal immune responses (1). The role of epithelial cells for direct elimination of *A. fumigatus* conidia has been discussed controversially (7). Several *in vitro* studies have indicated that epithelial cells may internalize and subject conidia to phagolysosomal degradation (8). In contrast, engulfment of conidia by bronchail epithelium has not been observed *in vivo* so far (9). More recently, eosinophils recruited in response to inhalative infection with *A. fumigatus* conidia were reported to contribute to fungal clearance in lung by soluble factors (10). Furthermore, eosinophils were demonstrated to generate both IL-17 and the CD4^+^ T helper cell type (Th)17 inducing cytokine IL-23 (11).

The family of ß2 integrins consists of four members and is formed by heterodimerization of an alpha subunit (CD11a-CD11d) with a common beta subunit (CD18) to form transmembrane receptors (12). The integrin receptor CD11b/CD18 (MAC-1) is primarily expressed by leukocytes of the myeloid lineage including monocytes/macrophages—which was name-giving (macrophage antigen 1, MAC-1)—but also by PMN, and conventional dendritic cells (DC). MAC-1 has been demonstrated to serve firstly as an adhesion receptor to various ligands including ICAM-1 which is necessary for transendothelial migration of macrophages and PMN (13). Secondly, it also operates as a major receptor for complement-opsonized pathogens, non-opsonized pathogens, and numerous serum factors (14) as well as a regulator of Fc receptor-mediated uptake of antibody-opsonized pathogens and immune complexes (15). Furthermore, MAC-1 serves as a negative regulator of DC- and macrophage-mediated T cell stimulation by binding to yet non-identified T cell receptors (16), and as a modifier of TLR-induced inflammatory signaling (17) and other signaling pathways (18). In accordance with the overall importance of ß2 integrins for immune responses, loss-of-function mutations of the CD18 gene result in the so-called leukocyte adhesion deficiency type 1 (LAD1) syndrome, a rare genetically determined disease (19). LAD1 patients suffer from severe, recurrent infections which require extensive treatment with anti-infective agents. Several studies have highlighted defective migration and phagocytosis of PMN as largely causative for rapid spreading of pathogens in LAD1 patients (20).

Recently, by using neutralizing antibodies MAC-1 dependent phagocytosis was identified as the relevant killing mechanism of *A. fumigatus* conidia by human PMN (21). This finding is in line with the observation that LAD1 patients often suffer from *A. fumigatus* infections. Here, we asked for the specific role of MAC-1 deficiency with regard to the clinical course in a mouse model of IPA, and focused on the early phase of infection to assess the role of MAC-1 for largely PMN-mediated antifungal immune response.

We show that CD11b^−/−^ mice display unaltered survival in IPA compared to WT mice. However, in the early phase of pulmonary infection lungs of CD11b^−/−^mice show a higher fungal burden which is associated with lower amounts of proinflammatory innate mediators like IL-1 in the bronchoalveolar lavage (BAL). In contrast, we detected elevated levels of the chemoattractant CCL5 in BALs derived from infected CD11b^−/−^mice, and enhanced bronchial infiltration of PMN. However, CD11b^−/−^PMN exerted an attenuated phagocytic uptake of *A. fumigatus* conidia *in vitro* which may explain the higher fungal burden in the lungs of infected CD11b^−/−^mice.

## Materials and Methods

### Fungal Strains and Cultivation Conditions

The *A. fumigatus* strain ATCC 46645 was cultured in *Aspergillus* minimal medium (AMM) with 1 % (w/v) glucose as described (6). Briefly, conidia were incubated on AMM agar plates for 4 days at 37°C and 5 % CO_2_. For preparation of spore suspensions, plates were washed with sterile water containing a small amount of glass pearls. The obtained spore suspension was filtered twice through a sterile 40 μm nylon mesh and stored in sterile water at 4°C.

### Mice

Female C57BL/6J mice and CD11b^−/−^ mice on C57BL/6 background [B6.129S4-Itgamtm1Myd/J; (22)] were obtained from Jackson Laboratory (Bar Harbor, ME) and were maintained in the Translational Animal Research Center of the University Medical Center Mainz under pathogen-free conditions on a standard diet. All animal procedures were performed in accordance with the institutional guidelines and approved by the responsible national authority (National Investigation Office Rhineland-Pfalz, Approval ID: AZ 23 177-07/G11-1-034).

### Mouse Model of Invasive Aspergillosis (IA)

Mice were anesthetized and challenged with 1 × 10^7^ *A. fumigatus* conidia intratracheally as further described (23). In brief, a 22G indwelling venous catheter (B. Braun AG, Melsungen, Germany) was inserted into the trachea and 100 μl sterile water or corresponding fungal suspension was administered through the catheter. To enhance dispersion in the lungs, mice were ventilated mechanically with 250 breaths/min, 300 μl/breath for 2 min using an animal respirator (MiniVent, Hugo Sachs, March-Hugstetten, Germany) as described (6). Severity of systemic infection was daily examined by the evaluation of weight, activity, posture, skin, and fur appearance as described by Prüfer and coworkers, and overall survival was monitored for up to 14 days. Mice with severe symptoms determined by clinical scores were immediately euthanized as required by the institutional animal ethics guide lines. Where indicated, PMN depletion was induced by i.p. injection of anti-Gr-1 antibody (150 μg, clone RB6-8C5) binding Ly6G^+^ PMN and Ly6C^+^ myeloid cells 1 day before infection.

### *In vivo* Ungal Killing

To characterize the fungal clearance of the lungs *in vivo*, some mice were sacrificed 24 h after infection. Lungs were removed, mechanically homogenized and serial dilutions were plated on Sabouraud-4 % Glucose agar (Carl Roth, Karlsruhe, Germany). After 24 and 48 h at 37°C and 5% CO_2_, colony-forming units (CFU) were counted.

### Histopathologic Analysis

Mice were euthanized 24 h after infection. Afterwards, mouse lungs were filled with 10% formalin via the trachea. Paraffin embedded blocks were prepared, sections (5 μm) were taken and stained with H&E to assess inflammatory responses. For analysis of inflammation H&E-stained tissue sections were examined by microscopy in a blinded fashion for peribronchial, perivascular and tissue inflammation, using a scoring system (0–4) on 5 randomly selected areas on each slide (24) with a BX40 microscope equipped with a CCD camera (Olympus, Hamburg, Germany).

### Flow Cytometric Analysis

Mice were sacrificed 24 h after infection. Blood samples were collected by retro-orbital incision and lungs were flushed with 1 ml PBS. Cells in the blood and bronchoalveolar lavage fluid (BALF) were analyzed by flow cytometry or an animal blood counter (Vet abc hematology analyzer, scil animal care, Viernheim, Germany), respectively. For analyses by flow cytometry, cells were washed in staining buffer (PBS/2% FCS), and Fc receptors were blocked by incubation with rat anti-mouse CD16/CD32 antibody (clone 2.4G2) for 15 min at room temperature. Then, cells were incubated with FITC-conjugated anti-Ly6C (HK1.4), and anti-MHCII (M5/114.15.2), Alexa Fluor 488-conjugated anti-MHCI (AF6-88.5.5.3), PE-Cy-7-labeled anti-CD11c (N418), anti-CD62L (MEL-14), anti-CD86 (GL-1), and anti-CD115 (AFS98), APC-conjugated anti-CD11c (N418), anti-CD68 (FA-11), and anti-MHCII (M5/114.15.2), and Pacific Blue-conjugated anti-F4/80 (BM8), and anti-Ly6G (1A8). Corresponding isotype control antibodies were used. All antibodies were obtained from Biolegend (San Diego, CA) or Thermo Fisher/eBioscience. Samples were analyzed using a flow cytometer (LSR II, Becton Dickinson, Heidelberg, Germany), and data were processed using FlowJo software V8.8.7 (Tree Star Inc., Ashland, OR, USA). The general gating strategy is shown in Figure S1.

### Cytospin Analysis

For detection of lung infiltrating PMN, 150 μl of BALF (see above) were transferred via cytospin (3,500 rpm for 5 min; Cytospin 3, Thermo Fisher) onto microscope slides, treated with the Diff Quick Staining Set (Microptic, Barcelona, Spain), air-dried, and fixed as recommended. Samples were analyzed using a BX50WI microscope, equipped with a CCD camera (Olympus, Hamburg, Germany). PMN were identified based on their characteristic segmented nuclei.

### Cytokine Detection

Amounts of cytokines in BAL and blood samples were quantified by Cytometric bead array using the mouse CBA flex sets following the manufacturer's instructions (BD Bioscience, San Jose, CA).

### Fungal Uptake by PMN

PMN were purified from C57BL/6 bone marrow by magnetic cell sorting (MACS) using biotin-labeled Ly6G specific antibodies and streptavidin-conjugated beads (Miltenyi, Bergisch Gladbach, Germany) according to the manufacturer's protocol. The cell purity (CD11b^+^Ly6G^+^) exceeded 90% as assessed by flow cytometry. PMN (10^6^ cells/ml) were incubated in Iscove's medium (Thermo Fisher Scientific, Waltham, MA, USA) supplemented with 5% (v/v) FCS, 2 mM l-glutamine, 50 μM ß-mercaptoethanol and 1 mM Na-pyruvate (SERVA Electrophoresis, Heidelberg, Germany) in 96-well plates (Greiner Bio One, Frickenhausen, Germany) with PE-fluorescent *A. fumigatus* conidia (25) germinated for 20 h at the indicated ratios in parallel at 4°C and 37°C to differentiate mere adhesion (4°C) from energy-dependent uptake (37°C). After 1 h the mean fluorescence intensity (MFI) was determined by flow cytometry. The general gating strategy is shown in Figure S5.

### Statistical Analysis

Statistical analysis was done with GraphPad Prism (version 5.0a for MacOS X; GraphPad Software, San Diego, CA, USA). Comparison of two different parameters was done using paired Student's *t*-test, and using one-way ANOVA when comparing more than two groups. For all analyses, p < 0.05 was considered as statistically significant.

## Results

### CD11b^−/−^ Mice Show Unaltered Survival of IPA but Increased Fungal Load and Decreased Pulmonary Inflammation

**Figure 1:**
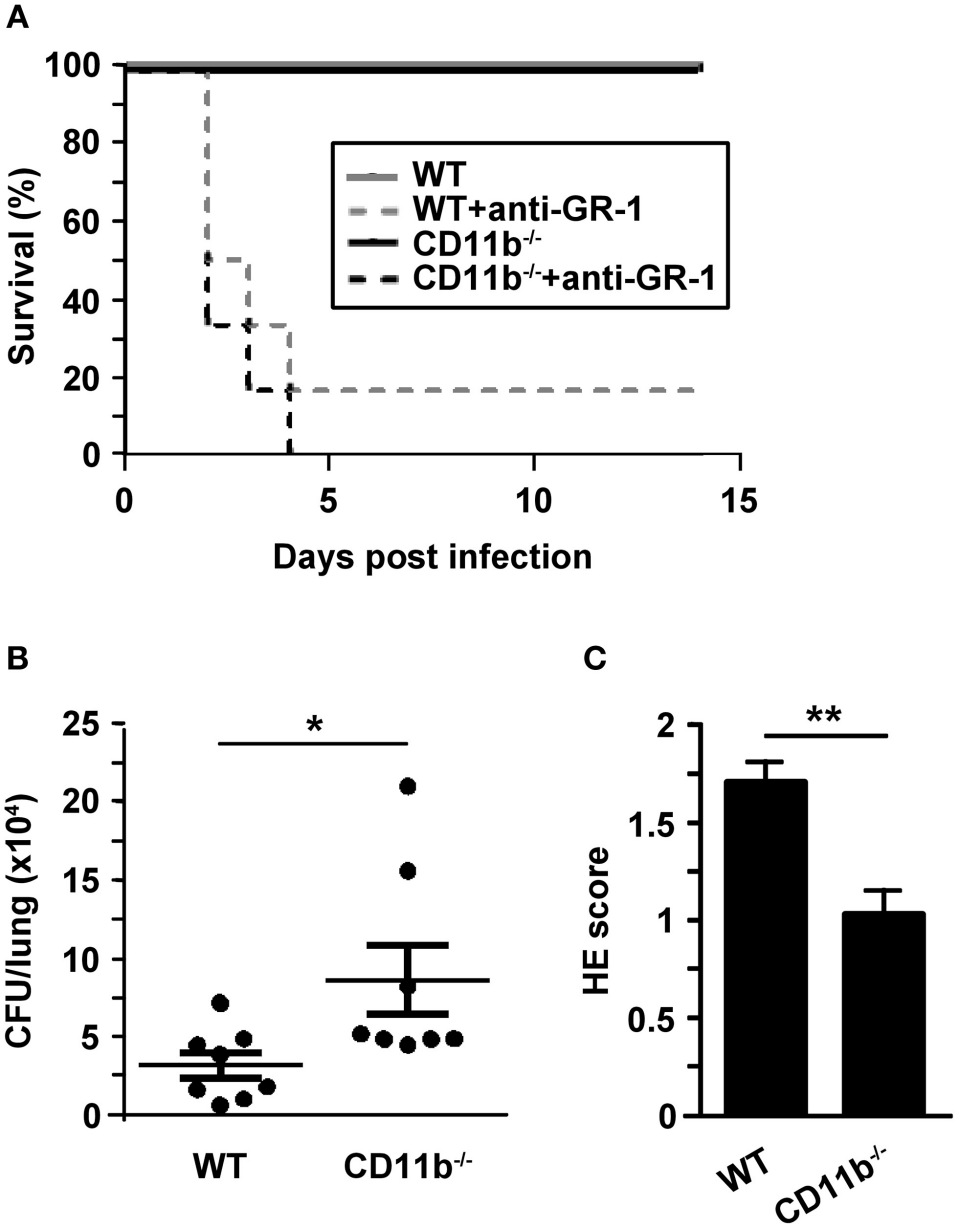
Invasive aspergillosis is not lethal for CD11b^−/−^ mice but lungs are characterized by attenuated inflammation, and higher fungal burden in an early phase after infection. Wild type (WT) and CD11b^−/−^mice were infected i.t. with *A. fumigatus* (each 10^7^ conidia/mouse) in 2 independent experiments. **(A)** The course of survival was daily monitored for 2 weeks and is presented in a Kaplan-Meier survival curve. In parallel settings in some mice PMN were depleted by injection of anti-GR1 antibody one day before infection. Data show the cumulative results of two independent experiments with a total of 7 (WT) or 8 (CD11b^−/−^) mice/group, and each 6 mice/PMN-depleted group). **(B,C)** 24 h after infection, mice were euthanized. **(B)** Paraffin sections of prepared lung sections were stained with H&E, and peribronchial and perivascular inflammation was scored. Data denote the mean ± SEM of 8 samples analyzed per group. **(C)** Serial dilutions of lungs homogenates were plated on agar plates for 2d, and CFU were counted. Data show the single data points, and the according mean ± SEM of 8 mice/group. **(B,C)** Statistically significant differences between groups are indicated (*p < 0.05, **p < 0.01).

To assess the relevance of MAC-1 for clearance of pulmonary infection with *A. fumigatus*, we observed the course of disease in wild type (WT) and CD11b^−/−^ mice. WT mice are well known to sufficiently clear IPA infection largely by activated PMN (6). In a group of animals an anti-GR-1 antibody was applied prior to infection with *A. fumigatus* (d0) to deplete primarily PMN as an internal control for success of infection in parallel settings. As depicted in Figure 1A, all non-depleted mice survived infection monitored over 2 weeks although the clinical signs were more aggravated in the first days after inoculation in case of CD11b^−/−^mice (not shown). As expected, PMN-depleted mice showed lowered survival underlining the essential role of PMN to limit spreading of *A. fumigatus* for survival of IPA. PMN-depleted CD11b^−/−^ mice displayed a somewhat lower tendency to survive as compared with WT mice.

Next, we focused on the course of the early innate mainly PMN-driven immune response toward *A. fumigatus* infection. For this, BAL fluid (BALF) and lungs of infected mice were analyzed in more detail. Despite unaltered long-term survival of *A. fumigatus* infected CD11b^−/−^mice, lung homogenates obtained 24 h after infection showed an enhanced fungal burden as compared to lungs from WT mice (Figure 1B). In contrast, lung tissue from infected CD11b^−/−^mice displayed impaired pulmonary inflammation as assessed by Hematoxilin&Eosin (H&E) staining (Figure 1C). Furthermore, the number of mucus-producing cells in bronchi of *A. fumigatus* infected CD11b^−/−^mice was decreased compared to cells in bronchi of WT mice (Figure S2).

**Figure 2:**
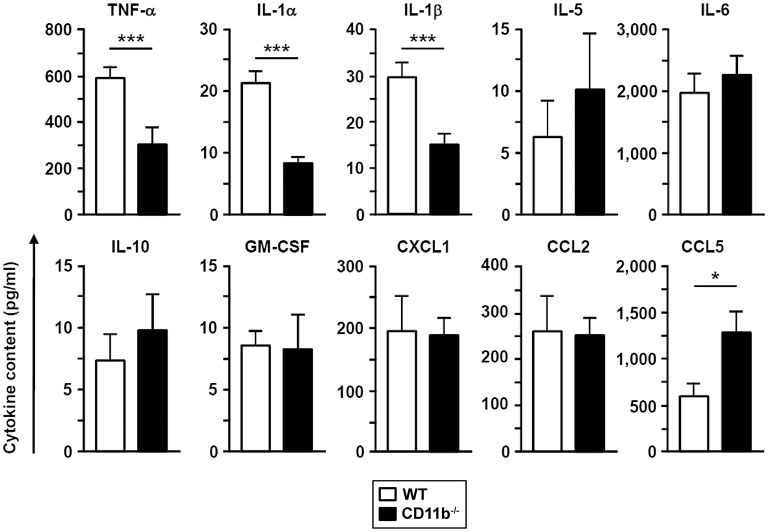
The BAL fluid of *A. fumigatus* infected CD11b^−/−^ mice contains lower levels of proinflammatory cytokines. WT and CD11b^−/−^ mice were infected i.t. with *A. fumigatus* as described in Figure 1. On the next day, mice were euthanized, and cytokines in BAL fluid were analyzed. Data denote the mean ± SEM of 4–8 samples analyzed per group. Statistically significant differences between groups are indicated (*p < 0.05, ***p < 0.001).

In accordance with diminished cellular lung inflammation, BALF derived from infected CD11b^−/−^ mice contained lower levels of three different proinflammatory cytokines (TNF-α, IL-1α, IL-1β) compared to BALF from WT mice while levels of other cytokines and chemokines (IL-5, IL-6, IL-10, GM-CSF, CXCL1, CCL2) were comparable (Figure 2). In contrast, BALF obtained from CD11b^−/−^mice showed higher levels of the chemokine CCL5 known as a relevant chemoattractant in innate and adaptive immune cells.

### Pulmonary PMN Infiltrates Are Increased in CD11b^−/−^ Mice Upon IPA

**Figure 3:**
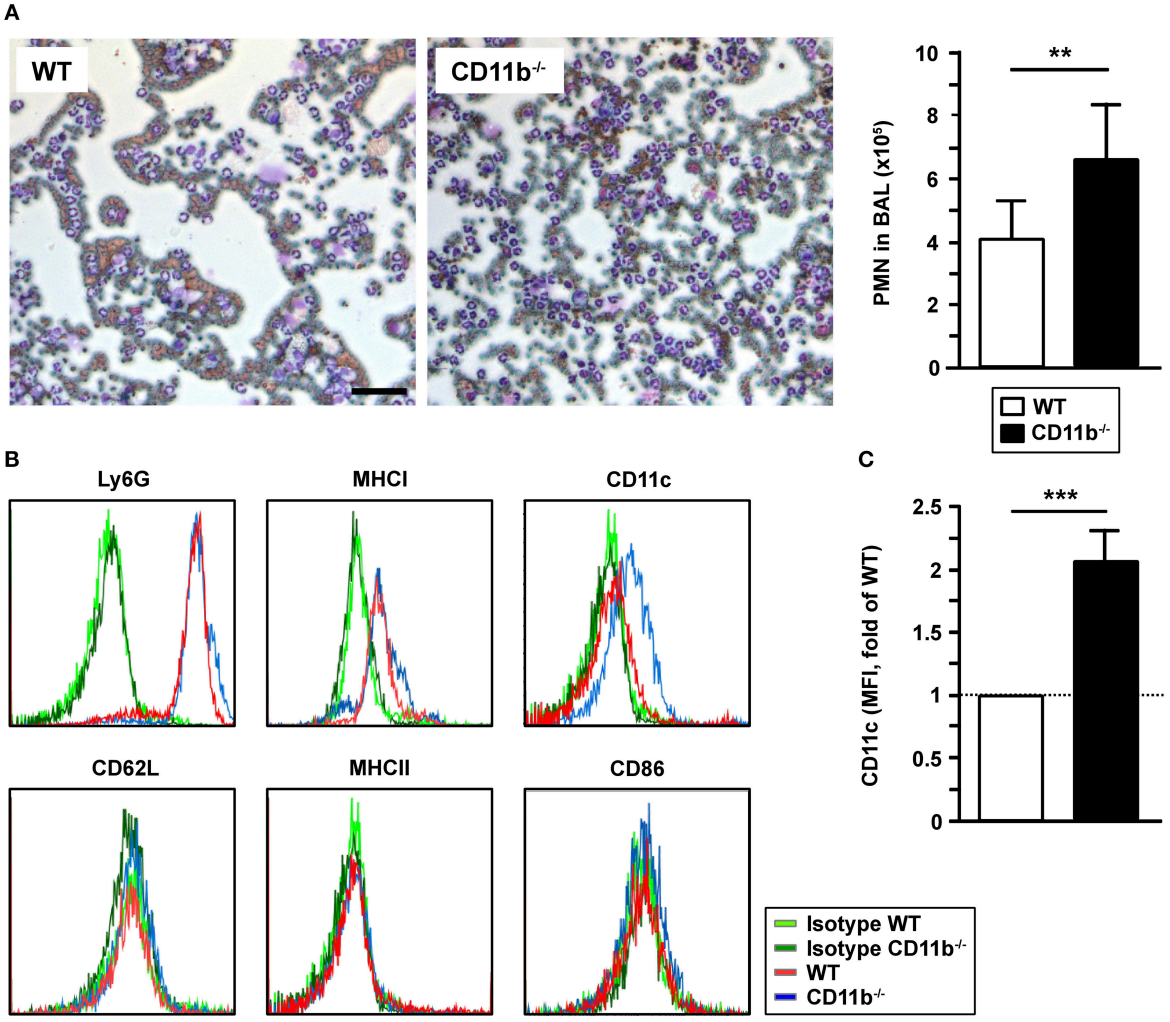
CD11b^−/−^mice infected with *A. fumigatus* are characterized by elevated lung infiltration of PMN. WT and CD11b^−/−^ mice were infected i.t. with *A. fumigatus* as described in Figure 1. One day after infection, mice were euthanized, and PMN were detected in BAL fluid. **(A)** 150 μl BAL fluid were cytospined, and stained with Diff Quick Staining Set® (Microptic). Left*:* Pictures were taken at 10x magnification, and stained PMN were counted. Magnification bar: 50 μm. Right*:* Quantification of PMN in BAL fluid. Date denote the mean mean ± SEM of 4 samples/group. **(B)** The immunophenotype of PMN in BAL fluid was assessed by flow cytometry. Histograms show the expression of surface markers of Ly6G+ cells and are representative of 4 samples per group. **(C)** Quantification of CD11c expression by Ly6G+ PMN in BAL fluid. Data denote the relative mean fluorescence intensities (MFI) ± SEM of 4 samples/group, normalized in each experiment on the MFI of WT Ly6G^+^ PMN. **(A,C)** Statistically significant differences between groups are indicated (**p < 0.01, ***p < 0.001).

In correlation with elevated CCL5 levels in BALF of infected CD11b^−/−^mice, we observed markedly higher numbers of PMN in the BALF of infected CD11b^−/−^mice compared to corresponding WT mice samples, whereas we observed no marked differences concerning other types of leukocytes (Figure 3A). Ly6G^+^ PMN derived from BALF expressed MHCI at comparable levels in both groups (Figure 3B). Interestingly, BALF-derived CD11b^−/−^Ly6G^+^ PMN expressed the mouse DC marker CD11c at moderate extent (Figures 3B,C). Infection-induced *de novo* expression of CD11c was reported for PMN in different mouse infectious disease models and was found to be associated with elevated expression of MHCII, CD86 and CD62L (26). However, lung-infiltrating Ly6G^+^ PMN of both genotypes expressed none of these receptors (Figure 3B).

Numbers of PMN, lymphocytes, and monocytes in the peripheral blood of *A. fumigatus* infected mice did not differ in a genotype-dependent manner (Figure S3A). Moreover, we observed no differences in the frequencies of cell lineage and activation marker expressing leukocytes in peripheral blood (Figure S3B, upper panel) and spleen (Figure S3B, lower panel). Ly6G^+^ PMN in peripheral blood and spleen were MHCI^+^. Moreover, some PMN fractions expressed CD62L in a genotype-independent manner (Figure S3C). In contrast to lung-derived PMN obtained from infected CD11b^−/−^mice (see Figure 3B), we observed no CD11c expression in PMN isolated from spleen and peripheral blood. A fraction of splenic Ly6G^+^ PMN expressed MHCII and CD86 not related to the genotype while Ly6G^+^ PMN in blood were deficient for these activation markers. In contrast to our analysis of BALF (see Figure 2), levels of IL-1 and chemokines in peripheral blood were largely comparable between *A. fumigatus* infected WT and CD11b^−/−^mice (Figure S4).

### Phagocytic Activity of CD11b^−/−^PMN Is Less Effective

**Figure 4:**
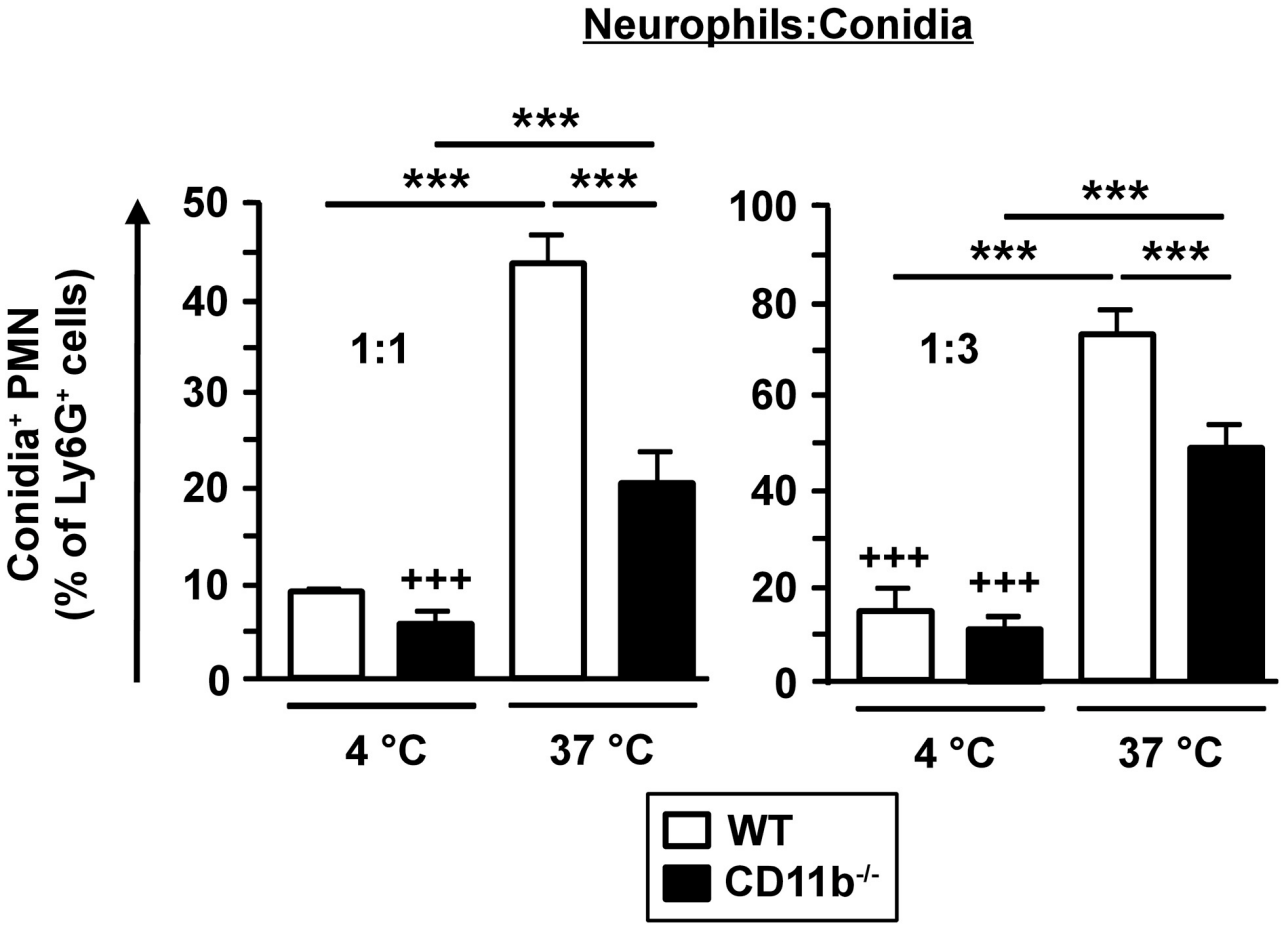
Phagocytosis of fungal conidia is less effective in CD11b^−/−^ PMN. PMN were purified from C57BL/6 bone marrow by magnetic cell sorting (MACS) using Ly6G-specific antibodies. PMN were co-incubated with PE-fluorescent *A. fumigatus* conidia at 37°C at ratios of 1:1 and 1:3, respectively. Simultaneous co-incubation at 4°C served as a negative control. After 1 h the frequency of PE-positive PMN was determined by flow cytometry. Data represent the mean ± SEM of 3 samples analyzed per group. Statistically significant differences between groups are indicated (***p < 0.001).

CD11b^−/−^ PMN were able to infiltrate *A. fumigatus* infected lungs, but were less capable to limit fungal spreading. As phagocytosis is the major effector mechanism of PMN to clear *A. fumigatus* conidia, we analyzed purified bone marrow derived Ly6G^+^ PMN to assess potential genotype-dependent differences in phagocytic capacity. Therefore, isolated PMN were incubated with fluorescent *A. fumigatus* conidia and were analyzed afterwards by flow cytometry. As depicted in Figure 4, only low fractions of PMN bound conidia at 4°C in a genotype-independent manner. However, as reflected by the parallel setting at 37°C to monitor energy-dependent internalization, CD11b^−/−^PMN showed lower phagocytic activity than WT PMN, indicating an impaired capability to clear *A. fumigatus*. Nevertheless, CD11b^−/−^mice were characterized by similar long term survival after IPA induction as WT mice (see Figure 1A).

## Discussion

The overall importance of ß2 integrin family members for immunological functions is highlighted by the severe immuno-compromised state of LAD1 patients (19). PMN are the first line of defense in the lung to prevent spreading of inhaled pathogens (27), and were demonstrated to require MAC-1 both for transendothelial migration (13) and phagocytosis of opsonized pathogens (5). Due to the frequent observation of IPA in LAD1 patients and the importance of MAC-1 for PMN effector functions, our aim was to investigate the specific role of MAC-1 in the early innate immune response to IPA which is largely mediated by PMN (6).

We found that survival of *A. fumigatus* infected CD11b^−/−^mice is not impaired after induction of IPA which suggests that MAC-1 despite its pronounced immunological functions is not critical for long term control of IPA. However, in the early phase of infection when spreading of *A. fumigatus* is mainly controlled by PMN (27) but also eosinophil (10, 11) effector mechanisms, several differences were observed in BALF and lungs derived from *A. fumigatus* infected CD11b^−/−^and WT mice. After the first 24 h, lungs derived from CD11b^−/−^mice showed enhanced fungal burden and lower bronchial inflammation despite higher numbers of PMN but not eosinophils in BALF derived from CD11b^−/−^mice. PMN are activated by various danger signals and upon contact with pathogens they contribute to the inflammatory response in infected tissue by secreting a variety of innate proinflammatory cytokines (28). PMN derived from BALF of *A. fumigatus* infected CD11b^−/−^mice produced lower levels of innate proinflammatory mediators like IL-1α, IL-1β, and TNF-α. In agreement, cellular inflammation in lung was attenuated as well. Accordingly, pulmonary fungal burden was increased. The diminished cellular inflammation as well as higher pulmonary pathogen burden despite elevated PMN infiltration was previously also demonstrated for CD11b^−/−^mice infected with *S. pneumoniae* (29).

In agreement with enhanced pulmonary fungal burden of *A. fumigatus* infected CD11b^−/−^mice we observed impaired phagocytic activity of CD11b^−/−^PMN toward opsonized *A. fumigatus* conidia compared to WT PMN. This observation is in line with the recent finding that MAC-1 is required in human PMN to kill *A. fumigatus* conidia by phagocytic uptake (21). As demonstrated by others, non-fungal pathogens like *S. pyogenes* are also efficiently bound via MAC-1 when opsonized by activated serum complement. Furthermore, cross-linking of MAC-1 results in NADPH oxygenate-dependent oxidative burst which is required by PMN for sufficient intracellular killing e.g., of *A. fumigatus* (30, 31) inducing apoptosis-like cell death in fungal conidia (32). Moreover, MAC-1 is necessary for Fc receptor-mediated phagocytic uptake of pathogens by neutrophils as shown for mouse CD11b^−/−^PMN and human PMN in CD11b blocking studies (33). Most recently, 2-(w-carboxyethyl)pyrrole which is generated due to the oxidative burst of activated PMN was demonstrated to modify MAC-1 ligands like fibrin resulting in enhanced affinity to MAC-1 on macrophages and a stimulation of macrophage migratory activity (34).

In several studies engagement of MAC-1 by pathogens as mimicked by cross-linking antibodies was shown to exert proinflammatory effects in PMN due to activation of members of the NF-κB transcription factor family, yielding elevated production of proinflammatory cytokines like TNF-α (35, 36). Therefore, the reduced activity of CD11b^−/−^ PMN to phagocytose *A. fumigatus* conidia and the impaired induction of an inflammatory milieu in lungs of *A. fumigatus* infected CD11b^−/−^ mice may be a consequence of attenuated PMN activation. Interestingly, other modes of CD11b activation as mediated by the more recently developed pharmacological activator leukadherin 1 were demonstrated to yield anti-inflammatory effects in myeloid cells which may be exploited for treatment of autoimmune diseases associated with hyperactive MAC-1 (37).

As mentioned above, BALF derived from CD11b^−/−^ mice contained higher numbers of PMN compared to BALF obtained from infected WT mice. This finding was unexpected as ß2 integrins were reported to be necessary to mediate firm adhesion of PMN to vessel endothelium as a prerequisite for PMN migration into extravascular space (38). However, by using lethally irradiated mice reconstituted with fetal liver cells from WT and CD18^−/−^mice Mizgerd and coworkers demonstrated that the requirement of CD18 for PMN infiltration depends on the type of pathogen used for infection (39). In case of intratracheal instillation of mice with *Escherichia coli*, LPS, or *Pseudomonas aeruginosa* only limited pulmonary infiltration of CD18^−/−^PMN was observed, while pulmonary infection with *S. pneumonia* caused PMN infiltration into the lungs in a CD18-independent manner. Comparable to that, LAD1 patients suffering from pneumonia were reported to display strong pulmonary PMN infiltration (40). Similar to our findings, CD11b^−/−^mice infected with *S. pneumoniae* also showed higher numbers of pulmonary PMN after 24h compared to infected WT mice (29), underlining that CD18 and even more specifically MAC-1 are not essential for PMN migration. Some studies have reported that LFA-1 may play a dominant role for transendothelial migration of PMN as evidenced in LFA-1 deficent mice (41). In addition, by applying blocking antibodies MAC-1 and LFA-1 were demonstrated to confer chemotaxis of PMN in a ligand-specific manner (42). While MAC-1 was required for migration toward fLMP (N-Formyl-Met-Leu-Phe), LFA-1 was required for IL-8 directed migration. Altogether, these studies confirm that MAC-1 expression is not essential for PMN migration *per se*.

MAC-1 not only contributes to migration and pathogen binding/phagocytosis by myeloid cells including PMN, but was also attributed to modulate PMN apoptosis. Coxon and coworkers reported that CD11b^−/−^ PMN isolated from the peritoneum after injection of thioglycollate were characterized by lower apoptosis than their WT counterparts (22). However, the contribution of MAC-1 signaling to apoptosis of activated PMN was discussed controversially. Zhang et al. reported that phagocytosis of pathogens by PMN promoted apoptosis of the latter which was associated with the induction of reactive oxygen species, and was enhanced by TNF-α (43). In contrast, CD11b^−/−^ PMN were not observed to undergo phagocytosis-induced apoptosis. Similar findings were reported for human PMN (44). On the contrary, Yan and coworkers showed that antibody-mediated blockade of ß2 integrins on human PMN elevated apoptosis after their activation by TNF-α or microbial stimuli (45). Further studies are necessary to elucidate the exact role of MAC-1 on PMN viability in case of IPA.

BALF obtained from infected CD11b^−/−^ mice contained higher levels of the chemokine CCL5 which is known to interact with the chemokine receptors CCR1, CCR3, CCR5, and CCR11, and thereby attracts many leukocyte populations (46). Early in the course of inhalative inflammation, CCL5 is generated by various activated cell types, including airway epithelial cells (47), airway smooth muscle cells (48), and lung fibroblasts (49). Moreover, *A. fumigatus* was reported to induce CCL5 in platelets (50), and activated PMN were demonstrated to produce CCL5 when incubated with *Toxoplasma gondii* (51). Therefore, further studies are required to elucidate which cell types are responsible for elevated CCL5 production in the lungs of *A. fumigatus* infected CD11b^−/−^mice. Mouse PMN express the CCL5-binding receptors CCR1 and CCR3 (52). Therefore, increased CCL5 levels in lungs of infected CD11b^−/−^mice may contribute to elevated PMN infiltration. Besides its leukocyte-attracting potency, CCL5 was demonstrated to contribute to the persistence of *A. fumigatus*-induced murine chronic allergic asthma (53). Interestingly, in this murine asthma model, airway hyperresponsiveness (AHR) was impaired in CCR5^−/−^mice. Moreover, diminished AHR was associated with attenuated peribronchial T cell and eosinophil infitration, and airway remodeling.

Our study focused on the role of CD11b during early innate responses toward inhalative infection with *A. fumigatus* which are primarily mediated by PMN (28), and as demonstrated more recently, also by eosinophils (10, 11). Additionally, PMN were shown to contribute to infiltration of CD11b^+^ conventional DC to lungs and mediastinal lymph nodes in IPA by activating CD11b^+^ DC in a cell-cell contact dependent manner via DC-SIGN (54). This C-type lectin receptor is expressed by DC and macrophages and mediates phagocytic uptake of *A. fumigatus* conidia (55). Moreover, DC and PMN were demonstrated to interact via DC-SIGN and MAC-1 (56). Therefore, MAC-1 on PMN may contribute to the activation of infiltrating DC in case of lung infections. In IA, activated DC produce IL-12 and IL-23 which induces Th1 (57) and Th17 (58) immunity. Moreover, IL-23 stimulates PMNs IL-17 production, and IL-17 induced ROS production by PMN (59), which contributes to fungal killing (5). Furthermore, infiltrating eosinophils constitute another source of IL-23 and IL-17 (11).

In conclusion, our study demonstrates, that CD11b deficiency on myeloid cells affects the early course of IPA. This may be due to the importance of MAC-1 for PMN effector functions, and their interplay with DC and other leukocytes (28). MAC-1 was demonstrated to contribute to eosinophil migration (60) and activation-dependent degranulation (61). Hence, we cannot rule out that this cell population may be affected by loss of MAC-1 which in turn may contribute to impaired fungal killing. Further work is also necessary to elucidate the long term course of IPA in CD11b^−/−^ mice with regard to the interplay of PMN with DC, the efficacy of adaptive immune responses, and the potential pulmonary overexpression of CCL5. Besides in patients with LAD1 syndrome, further evidence is needed to clarify whether our findings are relevant in other immunocompromised patients suffering from opportunistic infections.

## Author Contributions

DT, MR, and MB designed and supervised the study. DT, AC, and FR performed experiments, collected data, and analyzed the results. HB collected data, and analyzed the results. MB, MR, and DT wrote the manuscript. MT and SG revised the manuscript.

### Conflict of Interest Statement

The authors declare that the research was conducted in the absence of any commercial or financial relationships that could be construed as a potential conflict of interest.
