# Supplementary material for: CD11b Regulates Fungal Outgrowth but Not Neutrophil Recruitment in a Mouse Model of Invasive Pulmonary Aspergillosis
Source: Front Immunol. 2019 Feb 4;10:123. doi: 10.3389/fimmu.2019.00123 (PMC6369709; doi:10.3389/fimmu.2019.00123)
Supplement: Supplementary file 1 [file Data_Sheet_1.doc]

**Supplemental figures**


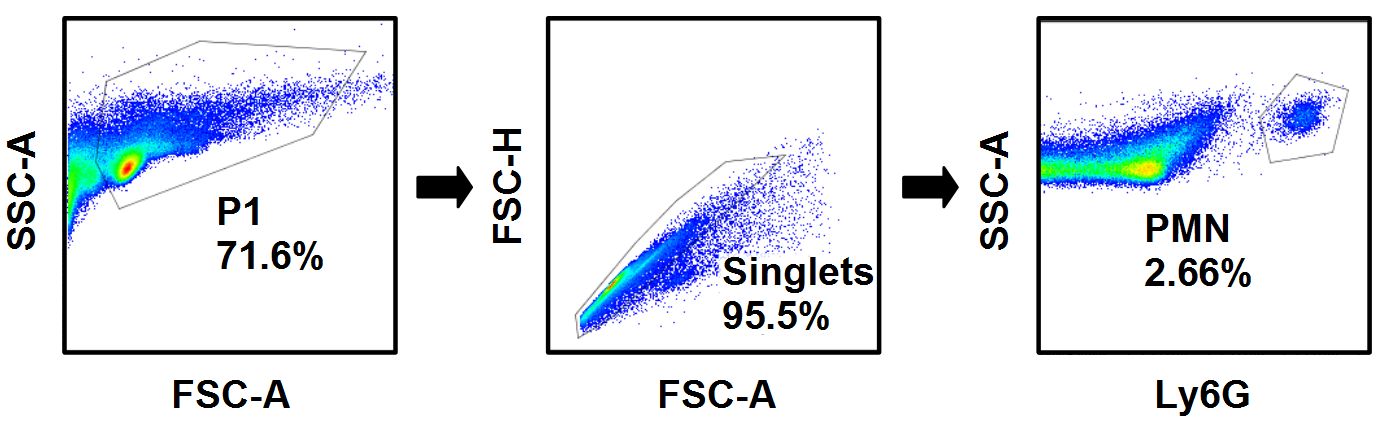


**Figure S1 |** Gating strategy for PMN analysis by flow cytometry. Left panel: exclusion of cell debris. Middle panel: exclusion of cell doublets. Right panel: gating on Ly5G^+^ cells (PMN lineage marker).


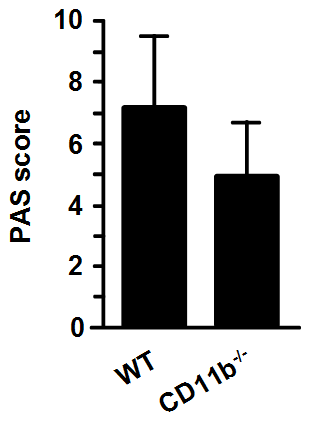


**Figure S2 |** Lungs of A. fumigates infected CD11b^-/-^ mice show lower frequencies of mucus-producing cells. WT and CD11b^-/-^ mice were infected i.t. with *A. fumigatus* as described in Figure 1, and were euthanized on the following day. Paraffin sections of prepared lung sections were stained by Periodic acid-Schiff reaction (PAS staining), and the number of mucus-producing cells per mm basal membrane was assessed. Data denote the mean ± SEM of 8 samples analyzed per group.


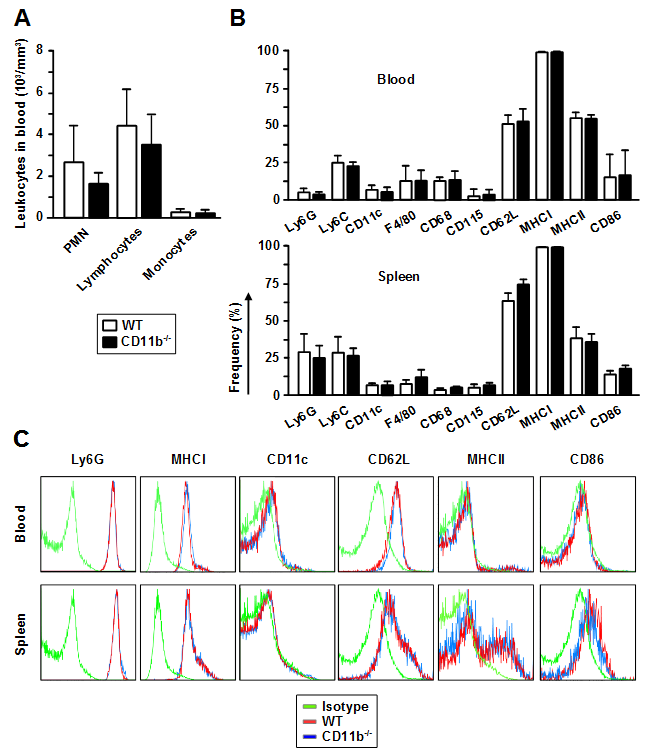


**Figure S3 |** Frequencies and immunophenotype of leukocytes derived from peripheral blood and spleens of *A. fumigatus* infected WT and CD11b^-/-^ mice are comparable. WT and CD11b^-/-^ mice were infected *i.t.* with *A. fumigatus* as described in Figure 1. One day after infection, mice were euthanized, and PMN were detected in BAL. (**A**) Leukocyte frequencies in blood were assessed using a Scil vet abc hematology analyzer (scil animal care company, Guernee, IL). Data denote the mean ± SEM of 8 samples/group. (**B**) Frequencies of immune cell populations, and the expression of activation markers in blood and spleen was assessed by flow cytometry. Data denote the mean ± SEM of 8 samples/group. (**C**) The immunophenotype of PMN in blood and spleen was analyzed by flow cytometry. Histograms show the expression of surface markers of Ly6G+ cells and are representative of 8 samples/group.


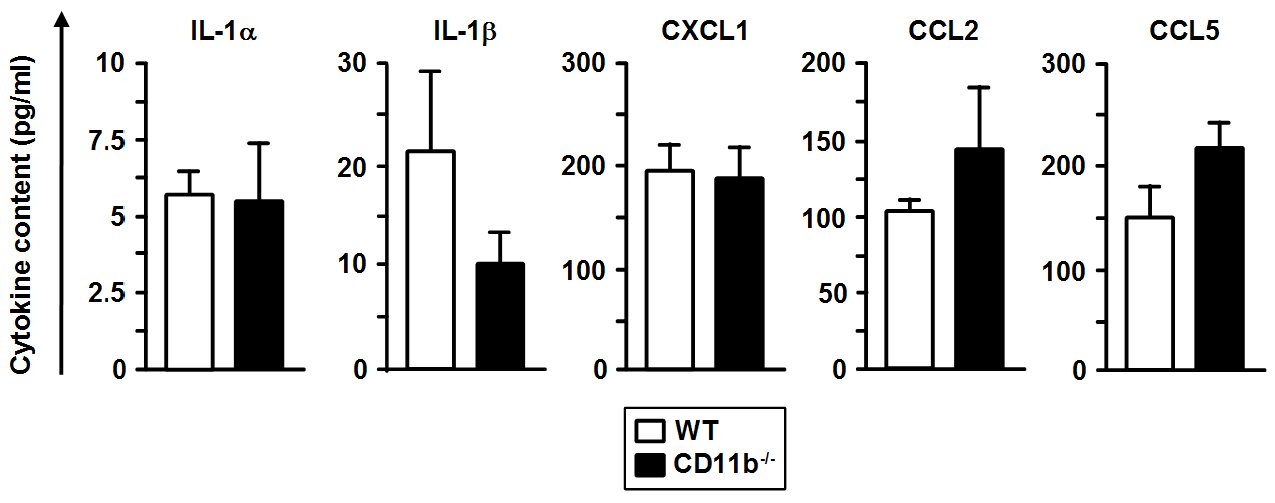


**Figure S4** **|** *A. fumigatus* infected WT and CD11b^-/-^ mice show comparable amounts of IL-1 and chemokines in peripheral blood. WT and CD11b^-/-^ mice were infected i.t. with *A. fumigatus* as described in Figure 1. On the next day, mice were euthanized, and blood contents of the indicated mediators were analyzed. Data denote the mean ± SEM of 4-8 samples analyzed per group.


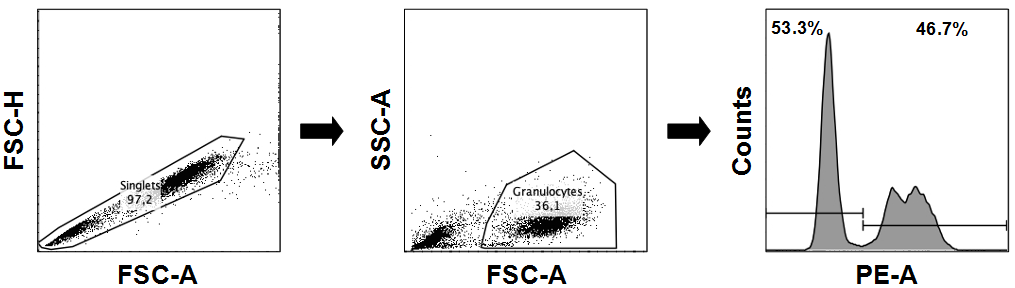


**Figure S5 |** Gating strategy for analysis of phagocytic activity of PMN incubated for 4h with PE-labeled *A. fumigatus* conidiae by flow cytometry. Left panel: exclusion of cell doublets. Middle panel: exclusion of cell debris. Right panel: quantitation of PE-positive PMN.
